# Association between asthma and headache: Findings from the NHANES 2001–2004

**DOI:** 10.1111/crj.13664

**Published:** 2023-07-10

**Authors:** Hok Leong Chin, Ka Kin Cheong

**Affiliations:** ^1^ Macau Private Clinic Macau Macau Special Administrative Region of China China; ^2^ Macao Academy of Medicine, Centro Hospitalar Conde de São Januário Macau Macau Special Administrative Region of China China

**Keywords:** asthma, asthma attack, epidemiology, headache, NHANES

## Abstract

**Introduction:**

With the adjustment of sociodemographic factors, our study aimed to explore the association between asthma control and headache using a representative sample in the United States.

**Methods:**

A total of participants aged >20 years from the National Health and Nutrition Examination Survey (NHANES) cycles 2001–2004 were included. The presence of asthma and headache was determined by questionnaires. Multivariate logistic regression was performed.

**Results:**

Participants with asthma had higher odds of suffering headaches (odds ratio = 1.62, 95% confidence interval: 1.30–2.02, *p* < 0.001). Those who had an asthma attack in the past year had higher odds of experiencing headaches than those who did not (odds ratio = 1.94, 95% confidence interval: 1.11–3.39, *p =* 0.022). No statistically significant association was found between participants who had emergency care visit for asthma in the past year and those who had not.

**Conclusion:**

Patients with asthma attack in the past year were more likely to have a headache than those who without.

## Graphical abstract

People with an asthma attack in the past year had higher odds of experiencing headaches than those who did not. No statistically significant association was found between people who had emergency care visit for asthma in the past year and those who had not.

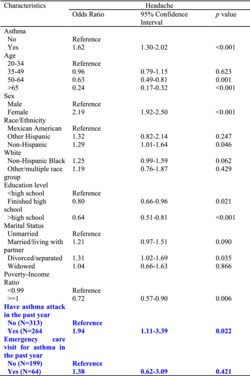

## 1 INTRODUCTION

Asthma and headache are both conditions that affect a significant population globally.^1^, ^2^ There are studies demonstrating possible overlapping between asthma and headache.^3^, ^4^, ^5^, ^6^, ^7^, ^8^, ^9^, ^10^, ^11^ With the use of a nationally representative dataset, our study aims to investigate the association between asthma and headache and whether the association varies across subgroups.

## 2 METHODS

### 2.1 Database and population

The National Health and Nutrition Examination Survey (NHANES) is a cross‐sectional national survey conducted by the Centers for Disease Control and Prevention. The survey covers about 5000 participants per year and is released every 2 years to represent the health status of the US population. A stratified multistage cluster sampling probability design is employed.^12^

This study used data from the 2001–2002 and 2003–2004 cycles as these are the cycles that contain headache information in adults. Eight thousand eight hundred and sixty‐one nonpregnant adults aged > = 20 years old with complete information on all independent and dependent variables were included.

### 2.2 Asthma

**FIGURE 1:**
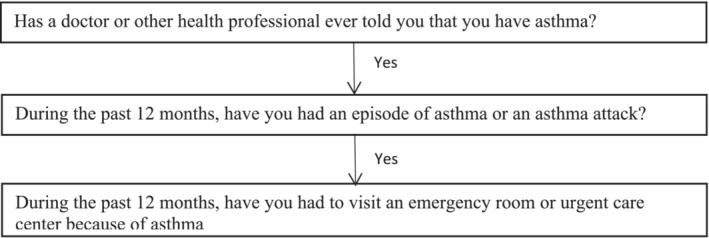
Asthma questions flow chart.

Asthma status was defined using the self‐reported questionnaire responses. Participants were considered to have asthma if they answered “yes” to the question “Has a doctor or other health professional ever told you that you have asthma?” For participants with asthma, the following questions were asked for further analysis: (1) “During the past 12 months, have you had an episode of asthma or an asthma attack?”, and (2) “During the past 12 months, have you had to visit an emergency room or urgent care center because of asthma?” Respondents who answered “don't know” would be excluded from the analysis of that specific question (Figure 1).

### 2.3 Headache

Headache status was defined using the self‐reported questionnaire responses. Headache was considered to be present if the participants answered “yes” to the question “During the past 3 months, did you have severe headaches or migraines?”

### 2.4 Covariates

The statistic model included sociodemographic characteristics as covariates: age, sex, race/ethnicity, education level, marital status, and poverty income ratio.

Age was divided into different age groups: 20–34 years, 35–49 years, 50–64 years, and >65 years. Sex was defined as male or female. Race/ethnicity was categorized as Mexican American, other Hispanic, non‐Hispanic White, non‐Hispanic Black, and other/multiple race group. Education level was divided into three groups: <high school, finished high school, and >high school. Marital status was categorized as unmarried, married/living with partner, divorced/separated, and widowed. The poverty–income ratio was split into those below the poverty line (<1) and those at or above the poverty line (> = 1).

### 2.5 Statistical analysis

All statistical analyses were performed using STATA/SE version 17.0 (Stata Corp, College Station, TX, USA). Appropriate sampling weight and masked variance units were applied. Logistic regression was performed to study the association between asthma and headache with adjustment for the sociodemographic covariates. Odds ratios (ORs) and 95% confidence intervals (95% CIs) were calculated. A *p*‐value <0.05 was considered statistically significant.

## 3 RESULTS

The mean age of the 8861 eligible participated individuals was 46.2 years (standard error = 0.37). 11.8% of study subjects had asthma. People with asthma status tended to be younger, female, other Hispanic, more educated, divorced/separated, and poorer (Table 1). 21.4% of study subjects had headache. People with headache were more likely to be younger, female, less educated, divorced/separated, and poorer (Table 2).

**TABLE 1:** Characteristics of participants in the National Health and Nutrition Examination Survey (NHANES) 2001–2004 according to asthma status.

Characteristics	*N* (%)	Without asthma (%)	With asthma (%)	*p* value
Total	8861 (100.00)	7917 (88.2)	944 (11.8)	
Age (years ± standard error)	46.2 ± 0.37	46.5 ± 0.39	44.0 ± 0.56	0.005
20–34	2086 (28.4)	1813 (24.4)	273 (3.9)	
35–49	2248 (33.3)	2027 (29.6)	221 (3.7)	
50–64	1861 (21.7)	1641 (19.1)	220 (2.6)	
>65	2666 (16.7)	2436 (15.2)	230 (1.6)	
Sex				0.001
Male	4616 (50.9)	4185 (45.7)	431 (5.2)	
Female	4245 (49.1)	3732 (42.6)	513 (6.6)	
Race/ethnicity				<0.001
Mexican American	1773 (7.4)	1660 (6.9)	113 (0.5)	
Other Hispanic	311 (4.6)	267 (3.9)	44 (0.7)	
Non‐Hispanic White	4778 (72.7)	4236 (63.9)	542 (8.8)	
Non‐Hispanic Black	1692 (10.8)	1484 (9.4)	208 (1.4)	
Other/multiple race group	307 (4.6)	270 (4.1)	37 (0.5)	
Education level				0.040
<High school	2696 (18.7)	2454 (16.8)	242 (1.9)	
Finished high school	2172 (26.3)	1959 (23.5)	213 (2.8)	
>High school	3993 (55.0)	3504 (47.9)	489 (7.1)	
Marital Status				0.046
Unmarried	1375 (17.1)	1207 (14.9)	168 (2.2)	
Married/living with partner	5352 (64.2)	4821 (57.1)	531 (7.2)	
Divorced/separated	1082 (11.9)	928 (10.1)	154 (1.8)	
Widowed	1052 (6.8)	961 (6.1)	91 (0.7)	
Poverty–income ratio				0.007
<0.99	1522 (12.9)	1337 (10.1)	185 (1.9)	
> = 1	7339 (87.1)	6580 (77.2)	759 (9.9)	

**TABLE 2:** Characteristics of participants in the National Health and Nutrition Examination Survey (NHANES) 2001–2004 according to headache status.

Characteristics	*N* (%)	Without headache (%)	With headache (%)	*p* value
Total	8861 (100.00)	7167 (78.6)	1694 (21.4)	
Age (years ± standard error)	46.2 ± 0.37	47.4 ± 0.44	41.7 ± 0.43	<0.001
20–34	2086 (28.4)	1564 (21.1)	522 (7.3)	
35–49	2248 (33.3)	1661 (24.7)	587 (8.6)	
50–64	1861 (21,7)	1516 (17.6)	345 (4.1)	
>65	2666 (16.7)	2426 (15.2)	240 (1.5)	
Sex				<0.001
Male	4616 (50.9)	3973 (43.0)	643 (7.9)	
Female	4245 (49.1)	3194 (35.6)	1051 (13.6)	
Race/ethnicity				0.325
Mexican American	1773 (7.4)	1408 (5.7)	365 (1.6)	
Other Hispanic	311 (4.6)	242 (3.5)	69 (1.2)	
Non‐Hispanic White	4778 (72.7)	3934 (57.6)	844 (15.1)	
Non‐Hispanic Black	1692 (10.8)	1335 (8.2)	357 (2.6)	
Other/multiple race group	307 (4.6)	248 (3.6)	59 (1.0)	
Education level				0.021
<High school	2696 (18.7)	2147 (14.1)	549 (4.6)	
Finished high school	2172 (26.3)	1742 (20.5)	430 (5.8)	
>High school	3993 (55.0)	3278 (44.0)	715 (11.0)	
Marital status				<0.001
Unmarried	1375 (17.1)	1074 (13.3)	301 (3.8)	
Married/living with partner	5352 (64.2)	4336 (50.5)	1016 (13.8)	
Divorced/separated	1082 (11.9)	831 (8.9)	251 (3.0)	
Widowed	1052 (6.8)	926 (5.9)	126 (0.9)	
Poverty–income ratio				<0.001
<0.99	1522 (12.9)	1132 (9.0)	390 (3.9)	
> = 1	7339 (87.1)	6035 (69.6)	1304 (17.6)	

Asthma was found to be associated with higher odds of headache among participants. A statistically significant OR of 1.62 was estimated, with a 95% CI between 1.30 and 2.02. A statistically significant positive association was found between female and headache, while a statistically significant negative association was found between education level and headache and between poverty–income ratio and headache. For participants who were considered to have asthma, those who had asthma attack in the past year had a higher odds of headache than those who had not. A statistically significant OR of 1.94 was estimated, with a 95% CI between 1.11 and 3.39. No statistically significant association was found between participants who had emergency care visit for asthma in the past year and those who had not (Table 3).

**TABLE 3:** Multivariable logistic regression model of asthma status and headache in the National Health and Nutrition Examination Survey (NHANES) 2001–2004.

Characteristics	Headache		
	Odds ratio	95% confidence interval	*p* value
Asthma			
No	Reference		
Yes	1.62	1.30–2.02	<0.001
Age			
20–34	Reference		
35–49	0.96	0.79–1.15	0.623
50–64	0.63	0.49–0.81	0.001
>65	0.24	0.17–0.32	<0.001
Sex			
Male	Reference		
Female	2.19	1.92–2.50	<0.001
Race/ethnicity			
Mexican American	Reference		
Other Hispanic	1.32	0.82–2.14	0.247
Non‐Hispanic White	1.29	1.01–1.64	0.046
Non‐Hispanic Black	1.25	0.99–1.59	0.062
Other/multiple race group	1.19	0.76–1.87	0.429
Education level			
<High school	Reference		
Finished high school	0.80	0.66–0.96	0.021
>High school	0.64	0.51–0.81	<0.001
Marital status			
Unmarried	Reference		
Married/living with partner	1.21	0.97–1.51	0.090
Divorced/separated	1.31	1.02–1.69	0.035
Widowed	1.04	0.66–1.63	0.866
Poverty–income ratio			
<0.99	Reference		
> = 1	0.72	0.57–0.90	0.006
Have asthma attack in the past year			
No (*N* = 313)	Reference		
Yes (*N* = 264)	1.94	1.11–3.39	0.022
Emergency care visit for asthma in the past year			
No (*N* = 199)	Reference		
Yes (*N* = 64)	1.38	0.62–3.09	0.421

## 4 DISCUSSION

Our study demonstrated an association between asthma and headache based on the data of NHANES 2001–2004 after the adjustment of covariates, representing the US population. Persons previously diagnosed with asthma were more likely to have headache than those without it. This finding supported the results of similar studies using different population‐based dataset from different countries.^3^, ^4^, ^5^, ^6^, ^7^, ^8^, ^9^, ^10^, ^11^ For example, the Head‐HUNT study showed that asthma patients were 1.5‐fold more possibly to have headache, both migraine and nonmigrainous, than people without asthma.^3^ A key contribution of our study was pointing out those who had asthma attack in the past year had higher odds to have headache but emergency care visit in the past year did not.

The underlying mechanism of association between asthma and headache has not been fully understood yet. However, degranulation of dural mast cells was found to prolong activation of trigeminal nerve.^13^, ^14^ Stimulation of the trigeminal nerve will release substance P, which causes neurogenic inflammation and contributes to the initiation of migraine.^7^, ^15^ Activation of mast cells also produces platelet‐activating factors and results in platelet aggregation during migraine.^16^ These vasoactive mediators were identified as causing bronchoconstriction and airway inflammation.^17^, ^18^

The major strength of our study was involving a large national representative dataset. The NHANES data were collected following structured guidelines with quality assurance. Different ethnic groups were included in a single study. Oversampling of certain population subgroups was done with weighting. This increased the generalizability and reliability of our study results. Additionally, other sociodemographic covariates that might alter the analysis results were taken into account.

Our study also contained some limitations. It is because of the cross‐sectional questionnaire design, a causal relationship between asthma and headache cannot be established and the types of headache cannot be differentiated. The asthma and headache status were determined by a self‐reported questionnaire, in which recall bias or measurement bias might also exist compared to other tools such as spirometry. Certain comorbidities were not included in this study due to their availability at NHANES database.

## 5 CONCLUSION

Our study demonstrated patients with asthma attack in the past year were more likely to have headache by using a large and diverse dataset from NHANES 2001–2004. Future studies can aim on characterizing the causality and temporality between asthma and headache onset.

## AUTHOR CONTRIBUTIONS

*Study conception and design*: Hok Leong Chin and Ka Kin Cheong. *Data analysis and interpretation*: Hok Leong Chin and Ka Kin Cheong. *Manuscript drafting*: Hok Leong Chin. *Table preparation*: Hok Leong Chin. *Critical revision of manuscript*: Hok Leong Chin and Ka Kin Cheong.

## CONFLICT OF INTEREST STATEMENT

No conflict of interest to be declared.

## ETHICS STATEMENT

The National Center for Health Statistics Research Ethics Review Board has approved NHANES cycles 2001–2004 (https://www.cdc.gov/nchs/nhanes/irba98.htm), Protocol #98‐12. Written informed consent was obtained from all participants (https://www.cdc.gov/nchs/nhanes/genetics/genetic_participants.htm).

## Data Availability

All data can be downloaded from the NHANES database (http://www.cdc.gov/nchs/nhanes.htm).
